# Introduction of SARS-CoV-2 variant of concern 20h/501Y.V2 (B.1.351) from Malawi to Italy

**DOI:** 10.1080/22221751.2021.1906757

**Published:** 2021-04-09

**Authors:** Federica Novazzi, Angelo Genoni, Pietro Giorgio Spezia, Daniele Focosi, Cristian Zago, Alberto Colombo, Gianluca Cassani, Renee Pasciuta, Antonio Tamborini, Agostino Rossi, Martina Prestia, Riccardo Capuano, Daniela Dalla Gasperina, Francesco Dentali, Paolo Severgnini, Walter Ageno, Cinzia Gambarini, Paola Stefanelli, Andreina Baj, Fabrizio Maggi

**Affiliations:** a Laboratory of Microbiology, ASST Sette Laghi, Varese, Italy; b Department of Medicine and Surgery, University of Insubria, Varese, Italy; c Department of Translational Research, University of Pisa, Pisa, Italy; d North-Western Tuscany Blood Bank, Pisa University Hospital, Pisa, Italy; e Internal Medicine Unit, ASST Dei Sette Laghi, Varese, Italy; f Anesthesia and Intensive Care, ASST Sette Laghi, Varese, Italy; g Biotechnology and Life Sciences Department, Varese, Italy; h S.C. Pneumology, ASST Dei Sette Laghi, Varese, Italy; i National Institute of Health, Rome, Italy

**Keywords:** SARS-CoV-2, COVID-19, variant of concern, B.1.1.351, 20h/501Y.V2, South African variant, VOC 202012/02

## Abstract

We report here an imported case of SARS-CoV-2 variant of concern B.1.1.351 (also known as 20H/501Y.V2 or “South African variant” or VOC 202012/02) in a 66-years old symptomatic male who returned from Malawi to Italy.

The SARS-CoV-2 variant of concern (VOC) 202012/02 (also known as B.1.351 in PANGOLIN phylogeny or 20H/501Y.V2 in NextStrain phylogeny) is currently causing a major outbreak of COVID-19 in South Africa. The South African variant harbours several spike mutations which in preliminary reports have been separately associated with escape from neutralizing monoclonal antibodies (mAbs) targeting either the N-terminal or the receptor-binding domain (RBD) [1], and from convalescent plasma collected during previous COVID-19 epidemic waves [2,3] (Figure 1).

**Figure 1.**
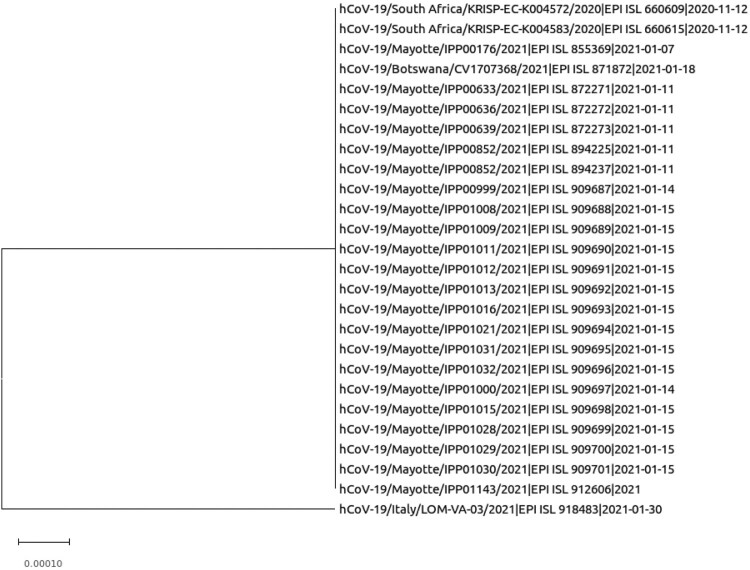
Phylogenetic tree of current SARS CoV-2 variant B.1.351.

Among the spike mutations, E484 K is considered the main driver of immune evasion to mAbs and convalescent sera [4] accordingly, many of the most potent mRNA vaccine-elicited mAbs were 3- to 10-fold less effective at neutralizing pseudotyped viruses carrying E484 K [5], which has unknown implications for protection.

We report here a 66-years old Italian male who tested positive for B.1.351. After returning from Malawi (Africa), on January 30 the man was admitted to the emergency department of Ospedale di Circolo e Fondazione Macchi, Varese with respiratory distress and fever (38°C). Considering the clinical symptoms, the nasopharyngeal sample (NPS) was collected for real-time PCR assay of SARS-CoV-2, and the patient was then admitted to the pneumology unit for a radiological picture of interstitial pneumonia. Here, the patient received continuous positive airway pressure ventilation with helmet first (FiO_2_ 50%, PEEP 7.5 cmH_2_O) and reservoir mask later. He was treated with low-molecular-weight heparin 6000 UI bid, vancomycin 500 mg every 6 h, and piperacillin/tazobactam 4.5 g every 8 h. At past medical history, he reported a congenital connective tissue disorder and both aortic and mitral valve biological replacement in 2016, for which he was under treatment with acetylsalicylic acid, bisoprolol, and furosemide. The patient was moved to ICU on Feb 2 for a worsening of respiratory pathology.

The NPS tested by the Alinity platform (Abbott) gave a positive result for SARS-CoV-2 RNA and the cycle threshold was 23. RT–PCR fragments corresponding to RBD in spike S gene of SARS-CoV-2 genome were amplified from purified viral RNAs by one-step RT–PCR using a QIAGEN OneStep RT–PCR Kit. Nested PCR reactions were named nPCRA and nPCRB and were carried out in 50 μl according to the manufacturer’s instructions. Amplification conditions were: 50°C for 30 min followed by 94°C for 15 min plus 40 cycles of 94°C for 30 s, 60°C for 30 s, and 72°C for 1 min with a final extension step of 72°C for 10 min. Following the first PCR reaction, 5 μL of amplified product was used for the second PCR reaction. Amplification conditions for nPCRB were: 95°C for 3 min plus 30 cycles of 95°C for 30 s, 60°C for 30 s and 72°C for 1 min with a final extension step of 72°C for 10 min. Amplified products were purified using QIAquick® PCR Purification kit (Qiagen, Manchester, UK) ready for Sanger sequencing analysis. Negative RNA samples and no-template controls were included in every assay and were always found to be negative. Sequences of the purified RT–PCR products were carried out using a BigDye Terminator v.1.1 cycle sequencing kit (Applied Biosystems). The sequencing reactions were purified using Centri-Sep Spin Columns (Princeton Separations, Adelphia, NJ) and analysed on a SeqStudio Genetic Analyzer (Applied Biosystems). Sequence variants were identified using CLC main workbench 7.0.0. The sequence of RBD was deposited in GenBank as MW560269, and GISAID as EPI_ISL_918483, and includes the B.1.1.351 barcoding mutations K417N, E484 K, and N501Y. The variant identification was confirmed by NGS sequencing of the whole viral genome, and deposited in GISAID as EPI_ISL_1012924.

Despite significant resistance to convalescent plasma and several mAbs, sera from human subjects vaccinated with mRNA-1273 led to 2.7 and a 6.4-fold geometric mean reduction in neutralization (but still 1:190) against K417N + E484K + N501Y + D614G or full B.1.351 Spike pseudovirus, respectively, when compared to the D614G VSV pseudovirus [5]. Similarly, sera from human subjects vaccinated with BNT162b2 led to 0.81- to a 1.46-fold geometric mean reduction in neutralization against an E484K + N501Y + D614G spike pseudovirus [6]. Finally, sera from persons vaccinated with one of 2 Chinese vaccines (BBIBP-CorV or recombinant dimeric RBD vaccine ZF2001) largely preserved neutralizing titres, with a slight reduction, against 501Y.V2 authentic virus [7].

As of Feb 3, 2021, 774 B.1.351sequences have been submitted from 25 countries across all continents (https://cov-lineages.org/global_report_B.1.351.html), but our own is the first from Italy. This finding confirms the risk of introduction from indirect flights if no surveillance measures are implemented at arrival. B.1.351-specific primer sets have been recently designed [8] and will facilitate large-scale screening programmes for this variant.
